# Supplementary material for: Barthelonids represent a deep-branching metamonad clade with mitochondrion-related organelles predicted to generate no ATP
Source: Proc Biol Sci. 2020 Sep 2;287(1934):20201538. doi: 10.1098/rspb.2020.1538 (PMC7542792; doi:10.1098/rspb.2020.1538)
Supplement: Table S3. BUSCO scores of the RNA-seq data of Barthelona sp. strain PAP020 and four fornicate species. [file rspb20201538supp3.docx]

**Table S3. BUSCO scores of the RNA-seq data of *Barthelona* sp. strain PAP020 and four fornicate species.**

| BUSCO score | *Barthelona* sp. strain PAP020 | *Carpediemonas membranifera* | *Aduncisulcus paluster* | *Dysnectes brevis* | *Kipferlia bialata* |
| --- | --- | --- | --- | --- | --- |
| SAR number | DRA009140 | SRR3734914 | SRR3741808 | SRR3742548 | DRR083618 |
| Complete (C) | 183 | 186 | 82 | 178 | 182 |
| Complete & single copy (S) | 141 | 141 | 61 | 175 | 159 |
| Complete & duplicated (D) | 42 | 45 | 21 | 3 | 23 |
| Fragmented (F) | 21 | 28 | 70 | 36 | 15 |
| Missing (M) | 99 | 89 | 151 | 89 | 106 |

Table S4. Comparison of the abundance of transcripts encoding putative MRO proteins among Barthelona sp. Strain PAP020, Dysnectes brevis, and Kipferlia bialata.
