# Supplementary material for: Barthelonids represent a deep-branching metamonad clade with mitochondrion-related organelles predicted to generate no ATP
Source: Proc Biol Sci. 2020 Sep 2;287(1934):20201538. doi: 10.1098/rspb.2020.1538 (PMC7542792; doi:10.1098/rspb.2020.1538)
Supplement: Table S4. Abundance of the transcripts encoding MRO protein candidates and acetyl-CoA synthases (ACS1-3) among Barthelona sp. Strain PAP020, Dysnectes brevis, and Kipferlia bialata. [file rspb20201538supp4.docx]

**Table S4. Abundance of the transcripts encoding MRO protein candidates and acetyl-CoA synthases (ACS1-3) among *Barthelona* sp. Strain PAP020, *Dysnectes brevis*, and *Kipferlia bialata*.**

|  | Transcript Per Million (TPM) | | |  |  | Transcript Per Million (TPM) | | |
| --- | --- | --- | --- | --- | --- | --- | --- | --- |
|  | PAP020 | *Dysnectes* | *Kipferlia* |  |  | PAP020 | *Dysnectes* | *Kipferlia* |
| Cpn60 | ND | 11 | 149.64 |  | Fdx | 12.53 | 15.26 | 203.26 |
| mtHsp70 | 1840.51 | 16.51 | 294.47 |  | Trx | 6978.7 | 4114.88 | 4897.22 |
| DnaJ | 114.68 | 174.68 | 561.47 |  | ME | 418.05 | 151.06 | 287.05 |
| GrpE | 2.65 | ND | ND |  | HydA | 373.5 | 39.34 | 37.52 |
| GCS L | 74.07 | 23.17 | 24.72 |  | PFO1 | 68.67 | 107.04 | 265.26 |
| GCS H | 1296.36 | 66.68 | 117.51 |  | PFO2 | 464.63 | 272.21 | 26.79 |
| GCS P | 157.26 | 99.08 | 155.87 |  | IscU | 14.55 | 137.87 | 549.02 |
| GCS T | 104.68 | 91 | 18.48 |  | IscS | 4.18 | 61.01 | 215.29 |
| SHMT | 99.63 | 31.33 | 63.62 |  | SCS-a | ND | ND | 273.08 |
| MGL | 964.34 | 24.55 | 187.67 |  | SCS-b | ND | ND | 369.37 |
| CS | 24.78 | 22.91 | 266.4 |  | ASCT1B | ND | ND | 194.62 |
| AlaAT | 120.51 | 79.26 | 83.09 |  | ASCT1C | ND | ND | 158.12 |
| AspAT | 501.41 | 146.4 | 396.59 |  | ACS1* | ND | 422 | 433.42 |
| GDH | 5504.01 | 4553.24 | 2772.51 |  | ACS2* | 717.63 | ND | ND |
| NuoE | 38.75 | 4.94 | 206.39 |  | ACS3* | 626.81 | ND | ND |
| NuoF | 24.98 | 43.6 | 159.54 |  | * ACS1, 2, and 3 are likely cytosolic enzymes. | | | |
