# Supplementary material for: Barthelonids represent a deep-branching metamonad clade with mitochondrion-related organelles predicted to generate no ATP
Source: Proc Biol Sci. 2020 Sep 2;287(1934):20201538. doi: 10.1098/rspb.2020.1538 (PMC7542792; doi:10.1098/rspb.2020.1538)
Supplement: Supplemental figure 1-6 [file rspb20201538supp5.pdf]

Supplemental figure 1

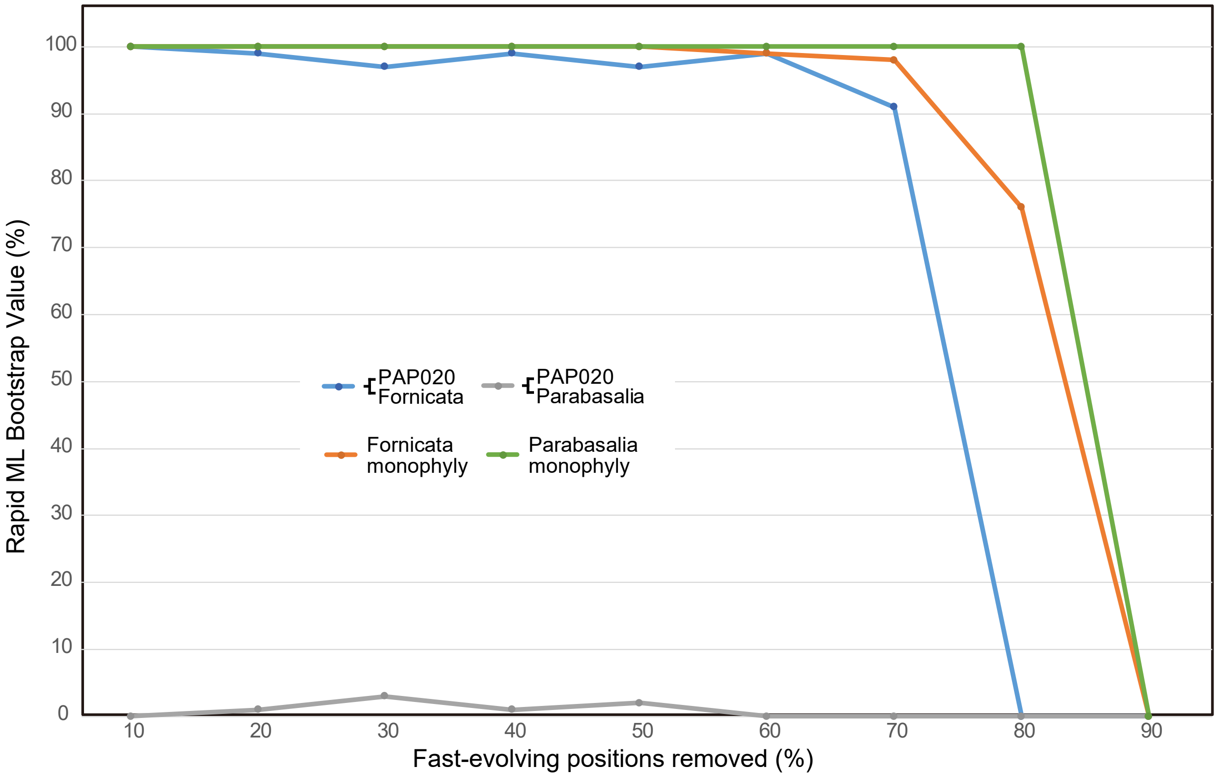

Figure S1: The impact of removal of fast-evolving alignment positions on the phylogenetic relationships among fornicates, parabasalids and *Barthelona* sp. strain PAP020.
Fast-evolving positions in the 148-gene alignments were progressively removed in 4,000 position increments. The filtered alignments were individually subjected to rapid ML bootstrap analyses using RAxML. For each data point, we plotted the support values for (i) the sister relationship between strain PAP020 and fornicates (blue), (ii) the monophyly of fornicates (orange), (iii) the sister relationship between strain PAP020 and parabasalids (gray) and (iv) the monophyly of parabasalids (green).

Supplemental figure 2

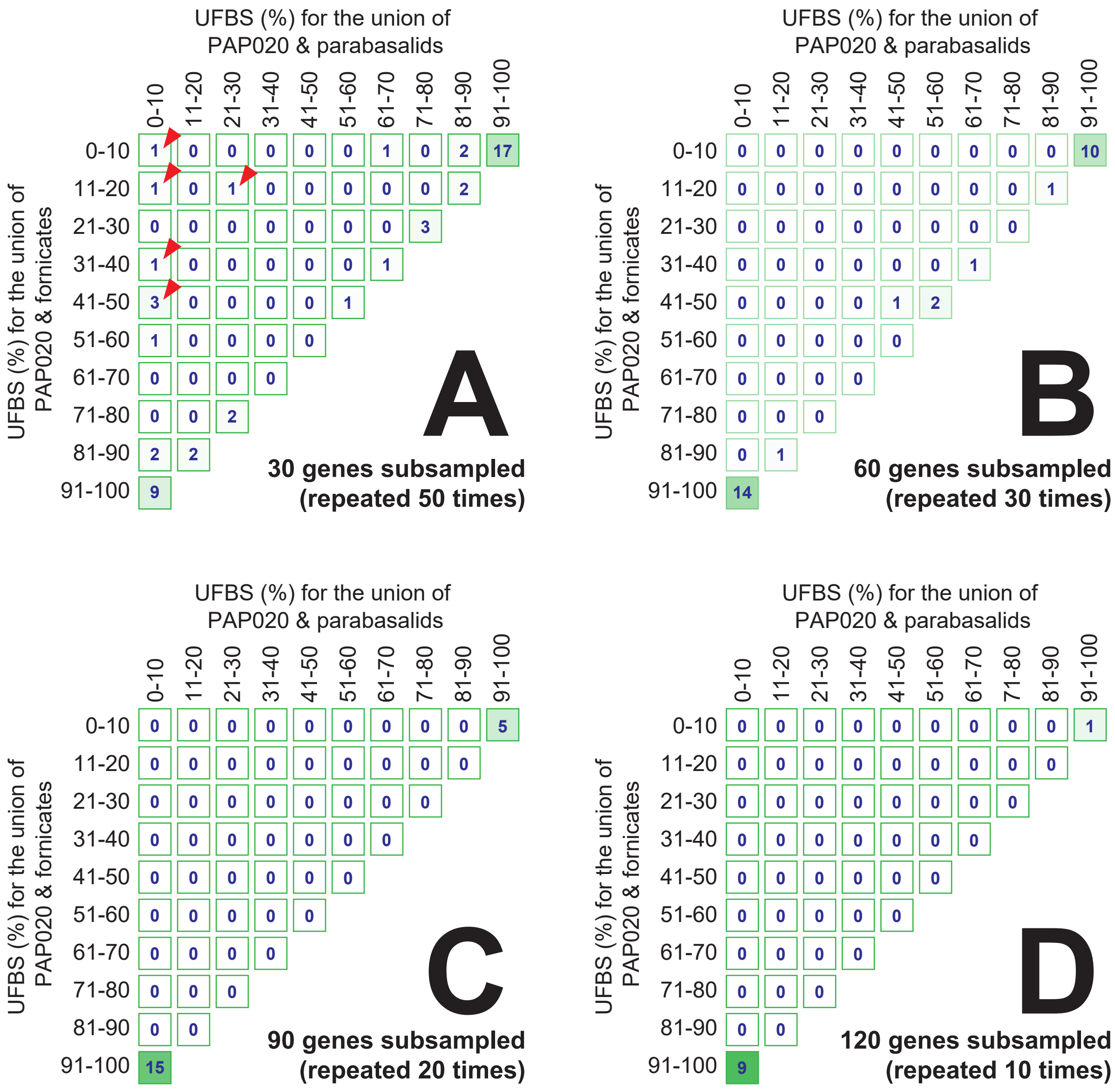

Figure S2: The impact of gene subsampling on the phylogenetic position of *Barthelona* sp. strain PAP020. A. 30 out of the 148 genes considered in the phylogenetic tree shown in Fig. 3 were randomly sampled, concatenated into a single alignment, and subjected to the ultrafast bootstrap analysis by using IQTREE. This procedure was repeated 50 times. After a close inspection of bootstrap trees, we noticed that strain PAP020 branched rarely with any eukaryotes except fornicates and parabasalids. Thus, the results from 50 bootstrap analyses were binned depending on the combination of the UFBS values for the union of parabasalids and strain PAP020 and that of fornicates and strain PAP020. For instance, the right-top bin indicates that the union of parabasalids and strain PAP020 received UFBS values greater than 90%, and the values for the union of fornicates and strain PAP020 ranged between 0 and 10%, in 17 out of 50 bootstrap analyses. In contrast, the left-bottom bin demonstrates that 9 out of the 50 bootstrap analyses supported the union of fornicates and strain PAP020 with UFBS values greater than 90%, while the union of parabasalids and strain PAP020 received only UFBS of 0-10%. The results of the ML analyses highlighted by red arrowheads are presented in Fig. S4. B. Summary of the bootstrap analyses of 30 alignments comprising 60 randomly sampled genes. C. Summary of the bootstrap analyses of 20 alignments comprising 90 randomly sampled genes. D. Summary of the bootstrap analyses of 10 alignments comprising 120 randomly sampled genes.

Supplemental figure 3

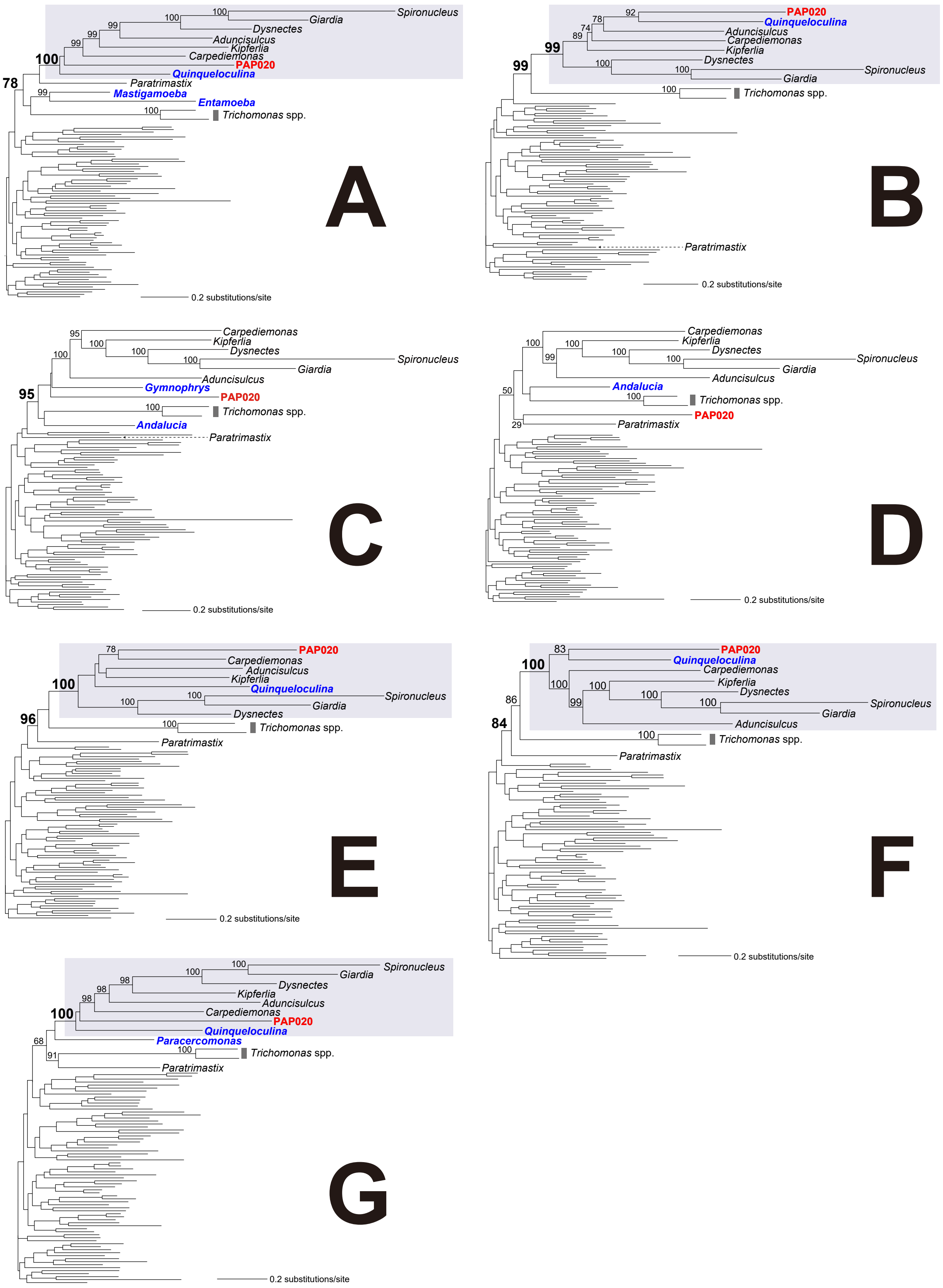

Figure S3: The results from the ML analyses of selected alignments comprising randomly sampled 30 genes.

The ML trees with UFBS values inferred from the alignments, which correspond to the data points highlighted by red arrowheads in Fig. S3A. We provide only the species names of Barthelona sp. strain PAP020 (PAP020 shown in red) and fornicates (black), as well as others that grouped with fornicates (blue). The UFBS values for the nodes, which are critical for the phylogenetic relationship among strain PAP020, metamonads, and others. A rhizarian Quinqueloculina sp. was found to form a clade with strain PAP020 and fornicates with UFBS values of 99 or 100% in A, B, E, F, and G (highlighted by shading). This grouping was not reconstructed from other alignments analyzed in gene subsampling analyses (See Figs. S3B-D).

Supplemental figure 4

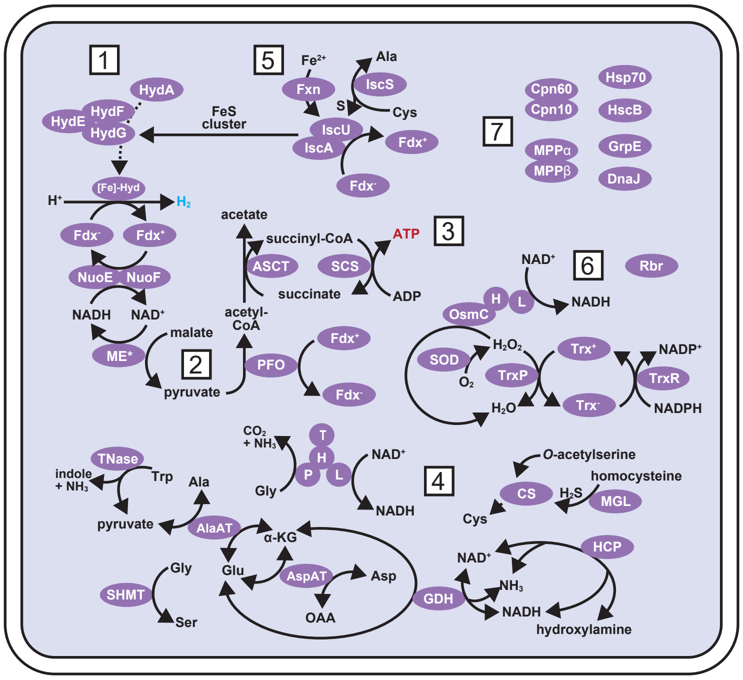

Figure S4: Metabolic pathways in the hydrogenosome in *Trichomonas vaginalis.* The details of this figure are the same as Fig. 4B. Purple ellipses represent the proteins localized in the hydrogenosome.

Supplemental figure 5

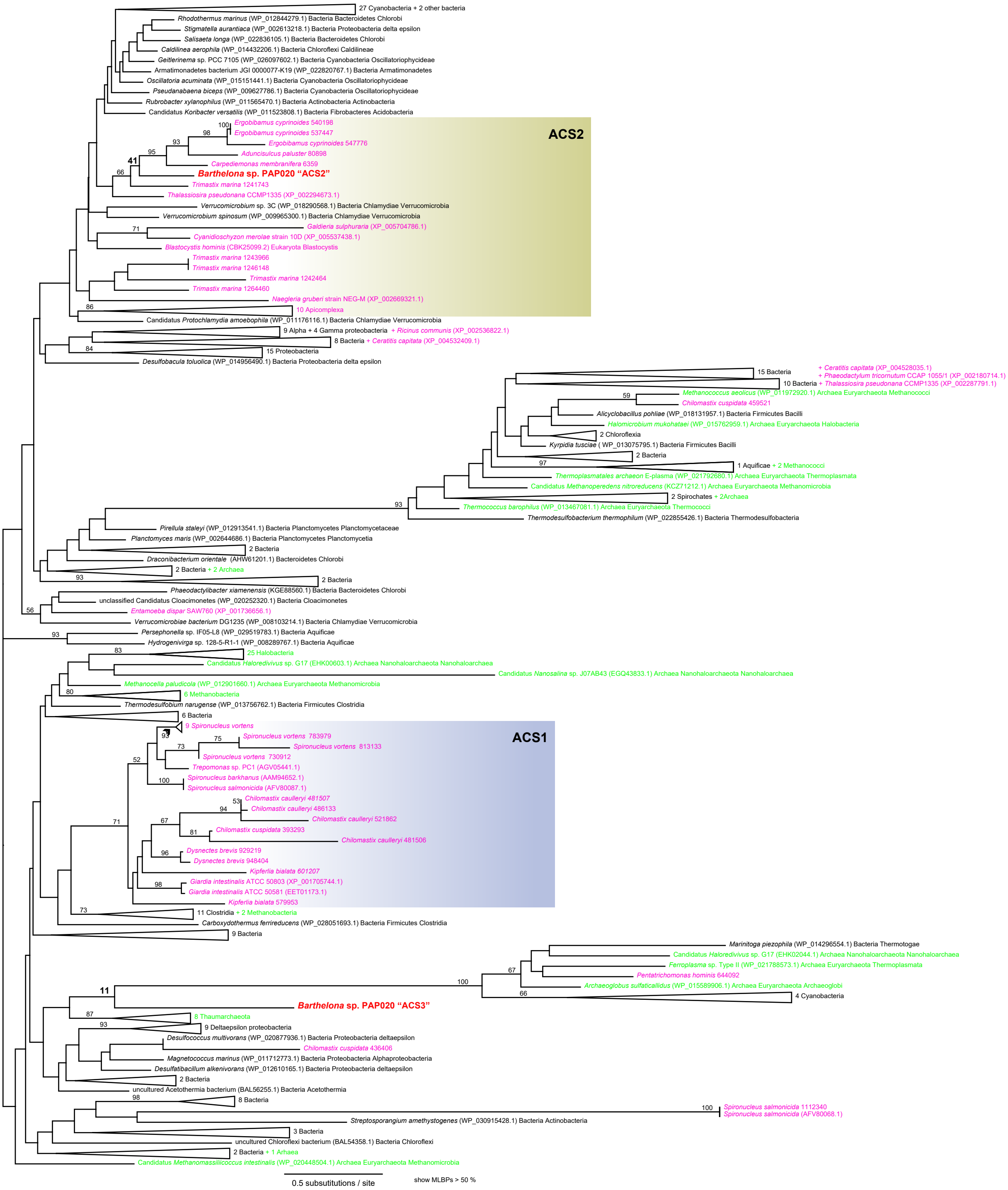

Figure S5: Phylogenetic tree of acetyl-CoA synthase (ACS) sequences.
The ACS phylogeny was inferred using the maximum-likelihood (ML) method and ML bootstrap values (MLBPs) were mapped on the ML tree. MLBPs below 50% are not shown. Two ACS sequences of *Barthelona* sp. strain PAP020 are highlighted in red. The pink- and green-colored sequences are of eukaryotes and archaea, respectively. The clades of ACS1 and ACS2 were defined by referring to the phylogenetic analysis presented in Leger et al. (2017).

Supplemental figure 6

A

```
ME1                              MIVNPLYNRPCHILVTGAAGAIGYNLAFQLADGGLLGKNQEIVLHLLEIPMGIERLEALV
ME2                              --MNPIYHRPCHILLTGASGTIGYNLVFQLANGGLLGANQEIVLHLLESPAKIDRLNALV
G. intestinalis  AAC47396.1      ------------------------------------------------------------
G. intestinalis  ESU42151.1      ------------------------------------------------------------
T. vaginalis     AAA91133.1      ------------------------------------------------------------
T. vaginalis     AAA87407.1      ------------------------------------------------------------
E. coli          VTQ44504.1      ------------------------------------------------------------

ME1                              MELEDSCCPLLRGVIATTDPQEAYTDVDIALLIGARPRGPGMQRKDLLVANGEIFKLAGN
ME2                              MELKDACCTLLRGIVATTDPQEAYGGIDIALLIGARARASGMLRKDLLVSNGEIFKLAGS
G. Intestinalis  AAC47396.1      ------------------------------------------------------------
G. Intestinalis  ESU42151.1      ------------------------------------------------------------
T. vaginalis     AAA91133.1      ------------------------------------------------------------
T. vaginalis     AAA87407.1      ------------------------------------------------------------
E. coli          VTQ44504.1      ------------------------------------------------------------

ME1                              YLNKYANDNCKVVVVGNPCNTNAIICASNAPKIPLGNFTALSRLDVNRARAKIAKMIESE
ME2                              YLNKYANEDCKVVVVGNPCNTNALICASNAPKIPLENFTVLSRLDVNRARAKLAEMIHHD
G. Intestinalis  AAC47396.1      ------------------------------------------------------------
G. Intestinalis  ESU42151.1      ------------------------------------------------------------
T. vaginalis     AAA91133.1      ------------------------------------------------------------
T. vaginalis     AAA87407.1      ------------------------------------------------------------
E. coli          VTQ44504.1      ------------------------------------------------------------

ME1                              VIDVENILIWGNHSATQVADITYAKYRANGQWKNVKEQMDLEYIEKEFIPFVASRGSAII
ME2                              VIDVENVIIWGNHSNTQVADVSYAKYRSKNEWKDVVGEVDLDFIERELIPFVASRGTVVL
G. Intestinalis  AAC47396.1      ------------------------------------------------------------
G. Intestinalis  ESU42151.1      ------------------------------------------------------------
T. vaginalis     AAA91133.1      ------------------------------------------------------------
T. vaginalis     AAA87407.1      ------------------------------------------------------------
E. coli          VTQ44504.1      ------------------------------------------------------------

ME1                              KKRGASSAGSAATAILGHTIDIINGSTPGCFTCMGVLTDGKVYGLPAGIFFSLPVICDGM
ME2                              KNRGASAAGSATAAIISHTDDLINGSKPGFFTCMGVFTDGIVYGLPAGLFFSLPVICDGM
G. Intestinalis  AAC47396.1      ------------------------------------------------------------
G. Intestinalis  ESU42151.1      ------------------------------------------------------------
T. vaginalis     AAA91133.1      ------------------------------------------------------------
T. vaginalis     AAA87407.1      ------------------------------------------------------------
E. coli          VTQ44504.1      ------------------------------------------------------------

ME1                              GKYVVIEDLEQPDWLRVKIEASAAELLEEREHADAFMGDRDFSDYRTEVCSLNDIYKHGK
ME2                              GKYVVIEDFEQCDWLREKIEASAAELIEEREQADAFMGDRDYSNYRAPTCSTNDVYKHGR
G. Intestinalis  AAC47396.1      ------------------------------------------------------------
G. Intestinalis  ESU42151.1      ---------------------------------------------------------MGS
T. vaginalis     AAA91133.1      -------------------------------------------MLASSVAAPVRNICRAK
T. vaginalis     AAA87407.1      -------------------------------------------MLTSSVSVPVRNICRAK
E. coli          VTQ44504.1      --------------------------------------------------MQFTHKKNRS

ME1                              VSHDFHDPQVLRDPAMNRDTAFTEIEREKLGIVGFLPPKVETIEQQVDRAYKSMHTLCDH
ME2                              VTHQ-HDPQVLRDPSMNRDTAFTEIEREQLGIVGFLPPKIETIEQQVDRAYKSMHTLCDH
G. Intestinalis  AAC47396.1      --MPITISTVLRNKDCNKDTAFTAAEREAHHIVARLPARVETIEQQISRCRAQFDVLTTP
G. Intestinalis  ESU42151.1      LKMPVTVSTVLRNKDYNKDTAFTDAERESHHIVARLPARVETLEQQISRCRTQFDALTTP
T. vaginalis     AAA91133.1      LPALKTGMTLLQDGDLSKGSAFTKEERDRLNLRGLLPYKVFTKDEQAARIRRQFELMPTP
T. vaginalis     AAA87407.1      VPTLKTGMTLLQDGDLSKGSAFTKEERDRLNLRGLLPYKVFTKDEQAARIRRQFELMPTP
E. coli          VTQ44504.1      LYIPYAGPVLLEFPLLNKGSAFSMEERSNFNLLGLLPEVVETIEEQAERAWIQYQGFKTE
```

B

```
PFO1                             ------------------VRGSRLLIMKFVACDANAAAAHISYACSDVSVIYPITPSTQM
PFO2                             ---------------------MQDRKPNYHSVDGNTAAIQAGYACSDLAFLYPITPSSPM
PFO3                             QRRVRGFFYHFYFYTILLKMSEPNNNHDYHGFDGNTAASHIAYFFSDNAIIYPITPSTPM
G. Intestinalis  EET01691.1      ------------------------MSVVHAPIDGCCAAAHVSYFFSDASVIYPITPSTPM
T. vaginalis XP_001582360.1      ---------------------MLRSFGKRIPGDGNTAATSVAYQLSETSFIYPITPATTM
T. vaginalis XP_001313671.1      ----------------MTLNQTTRFASKLVPMDGNAAAAHVAYNMSEASFLYPITPSTPM
E. coli WP      123038981.1      ----------------------------MITIDGNGAVASVAFRTSEVIAIYPITPSSTM
```

Figure S6: N-terminal alignments of malic enzyme (ME) and pyruvate:ferredoxin oxideoreductase (PFO) of *Barthelona* sp. strain PAP020

N-terminal amino acid sequence alignments of ME (A) and PFO (B) of *Barthelona* sp. strain PAP020, *Giardia intestinalis, Trichomonas vaginalis,* and *Escherichia coli.* The mature protein regions (shaded in blue) were predicted based on the *E. coli* sequences.
